# Characteristics and behavioral health needs of patients with patterns of high hospital use: implications for primary care providers

**DOI:** 10.1186/s12913-019-3894-7

**Published:** 2019-02-08

**Authors:** Karen G. Rentas, Laura Buckley, Dawn Wiest, Cortney A. Bruno

**Affiliations:** 1 Providence Health & Services, 11333 Sepulveda Blvd, Mission Hills, CA 91345 USA; 2 Camden Coalition of Healthcare Providers, 800 Cooper Street, 7th Floor, Camden, NJ 08102 USA; 3 Children’s Hospital of Philadelphia, 2716 South Street, Philadelphia, PA 19146 USA

**Keywords:** Primary health care, Care coordination, Integrated care, Complex care, Behavioral health, Substance use, Vulnerable populations

## Abstract

**Background:**

A small percentage of patients relies extensively on hospital-based care and account for a disproportionately high share of health care spending in the United States. Evidence shows that behavioral health conditions are common among these individuals, but understanding of their behavioral health needs is limited. This study aimed to understand the behavioral health characteristics and needs of patients with high hospital utilization patterns in Camden, New Jersey.

**Methods:**

The sample consisted of patients in a care management intervention for individuals with patterns of high hospital utilization who were referred for behavioral health assessments (*N* = 195). A clinical psychologist conducted the assessments, which informed a multiaxial evaluation with diagnostic criteria from the Diagnostic and Statistical Manual of Mental Disorders and a Mental Status Examination, to facilitate accurate diagnosis. Demographic characteristics, housing instability, exposure to trauma, and health care service utilization data were also collected through self-report and chart reviews.

**Results:**

Ninety percent of patients were diagnosed with a psychiatric and/or active substance use disorder. Depression was the most common psychiatric disorder and alcohol use was the most common substance use disorder. However, only 10% of patients with an active substance use disorder were in treatment, and only 17% of patients with a mental health diagnosis were receiving mental health treatment. Nearly all (91%) patients reported having a primary care provider at the time of assessment and most had seen their primary care provider within three months of their last hospital discharge. Non-medical barriers to health and wellness, specifically housing instability and exposure to trauma, were also common (35 and 61% of patients, respectively) among patients.

**Conclusion:**

Findings highlight the importance of identifying and treating patients with behavioral health needs in the primary care setting. Developing connections with community agencies who provide behavioral health and substance use treatment can enhance primary care providers’ efforts to address their patients’ non-medical barriers to treatment, as can embedding behavioral health providers within primary care offices. The study also underscores the need for trauma-informed care in primary care settings.

## Background

Primary care physicians (PCPs) play an important role in treating patients with high health care utilization patterns [1–3]. This small group of patients is gaining increasing attention as their care disproportionately accounts for health care expenditures in the United States [4–6]. In 2014, an estimated 1% of the population accounted for nearly 23% of total health care expenditures and 5% of the population accounted for just over 50% of overall health care spending [7]. Among patients under 65 with high hospital utilization rates, mental health and substance use disorders were among the top ten principal diagnoses for hospital stays [8]. Schizophrenia was the second most common diagnosis for those covered by Medicare or Medicaid, and alcohol related disorders was the sixth most common diagnosis for high-utilizing Medicaid patients [8]. Despite an increased focus on patients experiencing high hospital utilization, the nature and extent of their behavioral health complications are not well understood.

In the United States, individuals with mental illness have higher mortality rates than the general population, reaching a median of ten years of potential life lost [9]. Although certain behavioral health diagnoses do have higher mortality rates (e.g. psychosis, mood disorders), patients tend to die from the same chronic health conditions as the rest of the population, rather than from their mental illness [9]. Patients with high hospital utilization patterns are more likely to suffer from four or more chronic conditions than other patients [8]. Therefore, understanding and addressing their behavioral health needs and ensuring adherence to treatment for their chronic conditions are essential for this group. Moreover, the co-occurrence of a mental health disorder and medical chronic conditions has been associated with increased acute care utilization such as emergency department visits and hospital admissions when compared to individuals with medical chronic conditions alone [10].

Individuals with patterns of high acute care utilization may duly benefit from accessing primary care given the growing momentum in the US healthcare system towards behavioral health integration [11, 12]. The primary care setting offers a unique opportunity to address behavioral health needs. PCPs often see their patients near or during behavioral health crises. For example, a 2002 review article found that 45% of patients who died by suicide had seen their PCP in the month prior to their death [13]. Recent evidence also suggests PCPs may be uniquely positioned to screen for substance use and treat through brief interventions or refer to treatment given the frequency with which patients connect with these providers [14–17]. While there is growing recognition of the importance of understanding patients’ behavioral health needs for the provision of appropriate medical care, individual and system-level barriers often impede screening from occurring in the primary care setting [18].

### The Camden Coalition of Healthcare Providers’ focus on complex health and social needs

With a population of just under 77,000 residents, Camden is the largest urban center in southern New Jersey. The population is 49% Hispanic and 42% African American; 46% of residents speak a non-English language, predominantly Spanish. Camden perennially ranks among the most economically depressed cities in the country, with 4 in 10 individuals living below the federal poverty line [19]. And while violent crime has been on the decline in Camden, the rate per 1000 city residents is 9 times than for the state of New Jersey (19.66 vs. 2.29) [20, 21].

The Camden Coalition of Healthcare Providers (Camden Coalition) focuses on improving care for Camden residents who have complex health and social needs—a combination of multiple chronic conditions and social barriers to wellness—and reducing avoidable hospital readmissions. Patients enrolled in the Camden Coalition’s care management intervention work with an interdisciplinary team of nurses, social workers, and community health workers for an average of 90 days. Care teams use patient-centered, trauma-informed, and harm-reduction approaches, linking patients to primary care and other services in the community, and ultimately empowering patients to address their medical, behavioral, and social barriers to wellness upon “graduation” from the intervention. Annually, the Camden Coalition care management intervention enrolls an average of 20% of all patients identified through a triage process as eligible for the intervention.

To better understand the behavioral health of our patient population, we assessed mental health and substance use-related needs among a select group of enrolled patients, collecting information about mood, anxiety, psychotic, personality, and substance use disorders; childhood and adult trauma; suicidality; and mental health and substance use service utilization.

## Methods

### Sample and data collection

The purpose of the diagnostic assessment was to inform and improve care for patients enrolled in the Camden Coalition’s care management intervention. The care management intervention served individuals with a pattern of high hospital use who also demonstrated considerable social and medical complexity. The intervention sought to include patients whose hospital use may be mitigatable through care management.

To identify patients for the intervention, the Camden Coalition’s triage system incorporated a combination of Admit-Discharge-Transfer feeds and Electronic Medical Records (EMRs) from three local hospital systems and used both objective and subjective criteria. Patients who were assessed fit the eligibility criteria for the intervention: they were age 18–80; had health insurance coverage at intervention enrollment; had been hospitalized at least twice in the six months prior to enrollment; had two or more chronic conditions as documented in the EMR’s History and Physical Examination Write-Up from hospital admission, or past medical history from inpatient or outpatient encounter notes; and showed three or more “vulnerabilities” such as documented mental health comorbidity, evidence of difficulty accessing services, homelessness, active drug use, lack of social support, and/or taking more than five medications. Individuals were excluded from the intervention if their hospital admissions were unlikely to have been avoided, such as those related to oncology, planned surgical procedure (e.g. bariatric surgery), acute conditions without other complicating factors (e.g. appendicitis), and complications of a progressive chronic disease with limited treatments (e.g. multiple sclerosis or ALS). Individuals were also excluded if their index admissions were mental health-related only with no co-morbid medical conditions. Enrolled patients that presented with suspected behavioral health needs, or with self-reported behavioral health needs, were referred for a comprehensive behavioral health assessment by their care team.

Between September 2014 and January 2017, 225 enrolled patients were referred for assessment, of which 195 (87%) were available and agreed to be assessed for psychiatric disorders, substance use disorders, and experiences such as trauma and housing instability. The 195 patients who were assessed accounted for 38% of the patients active on the Camden Coalition’s care management panel at any point between September 2014 and January 2017. A bilingual Licensed Clinical Psychologist conducted face-to-face diagnostic assessments during home visits, in the community, or at patients’ appointments with other care providers. The psychologist explained the purpose of the assessment to patients who all spoke English and/or Spanish and who provided verbal consent prior to beginning the assessment. Not all patients who were assessed met criteria for a psychiatric or substance use disorder, but all are included in this study.

### Measures

#### Multiaxial evaluation

Data were collected using a comprehensive behavioral health assessment leading to a multiaxial evaluation with diagnostic criteria from the Diagnostic and Statistical Manual of Mental Disorders (DSM-IV) [22]. Each patient was assessed for psychiatric diagnoses, substance use disorders, and cigarette smoking. Psychiatric diagnoses included mood disorders (e.g., bipolar, depressive, dysthymic, unspecified), anxiety disorders (e.g., generalized anxiety, panic, posttraumatic stress), psychotic disorders (e.g., schizophrenia), and personality disorders (e.g. borderline personality disorder). Substance use disorders included diagnoses related to abuse, dependence, and remission from substances, including alcohol, cocaine, opioids, cannabis, sedatives/hypnotics, and amphetamines. Patients considered in full and/or partial remission either met none of the criteria or met only part, but not all, of the criteria for abuse or dependence over at least the last 12 months. Nicotine dependence was assessed and analyzed as a separate substance use variable. This was done in concordance with previous studies that have either excluded smoking status when looking at the impact of substance use disorders and emergency department frequent utilization or have analyzed it as a distinct variable [23–25]. Patients were also assessed for housing instability and history of trauma based on whether they experienced trauma before and/or after turning 18 years old.

Patients also underwent a Mental Status Examination (MSE), which is commonly used by mental health professionals, primary care doctors, and other clinicians to assess the patient’s behavioral and cognitive functioning to facilitate accurate diagnosis and clinical case formulation [26]. The MSE conducted for this study included direct observation and description of state-of-mind under the domains of appearance, attitude, psychomotor behavior, speech, affect, mood, thought process and content, perception, orientation, memory and concentration, and insight and reliability.

#### Patient characteristics

Demographic information and other characteristics (e.g., history of mental health treatment, substance use treatment, housing instability) were obtained through self-report and chart reviews.

#### Health care service utilization

Patients’ hospital records were reviewed to identify the number of hospital admissions and emergency department visits in the six months prior to enrollment in the intervention. Additionally, the records were reviewed to identify the number and type of chronic medical conditions at the time of enrollment, excluding psychiatric and substance use disorders.

## Results

### Patient characteristics

Table 1 summarizes data on patient demographics, relationship status, trauma exposure, and other social correlates of health. Eighty percent of patients reported being single, widowed, or separated/divorced at the time of enrollment. A slight majority were African-American (54%); an additional 30% were Hispanic/Latino. One-half of patients had no high school diploma or GED at the time of enrollment. A majority (61%) of patients reported experiencing trauma, which could include physical abuse, sexual abuse, emotional abuse, physical and emotional neglect, domestic violence, and witnessing violence, during childhood, adulthood, or both. In addition, one-third (35%) reported experiencing housing instability at time of assessment. These patients either reported homelessness, were living with friends or family due to financial stress, were staying at a shelter or rooming house, and/or had recently received an eviction notice.

**Table 1:** Patients characteristics

Characteristic	N (%)
Gender	
Female	98 (50)
Male	96 (49)
Transgender	1 (< 1)
Age at time of assessment (years)	
18–40	26 (13)
41–55	68 (35)
56–65	65 (33)
> 65	36 (19)
Race/Ethnicity	
Black/African American	106 (54)
Hispanic/Latino	59 (30)
White Non-Hispanic	28 (14)
Unknown	2 (1)
Marital status	
Single	103 (53)
Married/Domestic Partnership	38 (20)
Separated/Divorced	32 (16)
Widowed	21 (11)
Unknown	1 (< 1)
Highest education level completed	
No high school Diploma	103 (53)
High school graduate or GED	57 (29)
Some college but no Degree	18 (9)
Associate’s or Bachelor’s Degree	11 (6)
Graduate Degree	3 (< 2)
Unknown	3 (< 2)
Trauma Exposure	
None reported	76 (39)
Trauma exposure before age 18	36 (18)
Trauma exposure at age 18 or after	31 (16)
Trauma exposure before age 18 and after	52 (27)
Unstable Housing	69 (35)

### Health care profile and utilization prior to intervention enrollment

Table 2 displays data on health care utilization and medical chronic conditions for the 195 patients. More than one-half (58%) of patients had five or more chronic medical conditions, with hypertension being the most prevalent (75%). In the six months prior to intervention enrollment, 91% of patients had between two and four hospitalizations, 9% had five or more hospitalizations, and 37% of patients had five or more emergency department visits. Nearly all patients (91%) indicated they had a primary care provider (PCP), and two-thirds (66%) reported seeing their PCP within three months of their last hospitalization. Since having insurance is a requirement to participate in the intervention, all patients were insured. A slight majority of patients (56%) were Medicaid-only beneficiaries, with an additional 22% covered under both Medicare and Medicaid.

**Table 2:** Patient health care utilization and chronic medical conditions at time of intervention enrollment

Characteristic	N (%)
Timing of last visit with primary care provider (PCP)	
No PCP 30 or fewer days prior to intervention enrollment 30–90 days prior to intervention enrollment More than 90 days prior to intervention enrollment	18 (9) 69 (35) 61 (31) 46 (24)
Insurance	
Medicare only	26 (13)
Medicaid only	110 (56)
Dual Medicare Medicaid	43 (22)
Private (only or combo)	16 (9)
Number of inpatient admissions 6 months prior to intervention enrollment	
2	140 (72)
3–4	38 (19)
5 or more	17 (9)
Number of emergency department visits 6 months prior to intervention enrollment	
0–2	64 (33)
3–4	59 (30)
5 or more	72 (37)
Number of chronic medical conditions	
2	29 (15)
3–4	53 (27)
5 or more	113 (58)
Top five chronic medical conditions	
Hypertension	146 (75)
Asthma	95 (49)
Hyperlipidemia	93 (48)
Diabetes	88 (45)
Congestive heart failure	56 (29)

### DSM-IV diagnoses

Table 3 summarizes DSM-IV diagnoses and comorbidity patterns that resulted from the diagnostic assessments. Overall, 90% of patients had a psychiatric and/or active substance use disorder at the time of assessment. Eighty-three percent of patients met criteria for one or more psychiatric disorders, 17% of whom were engaged in treatment for their psychiatric disorder at the time of the assessment. Mood disorders were the most prevalent class (74%). The most prevalent psychiatric disorders were major depressive disorder (45%), bipolar disorder (15%), and posttraumatic stress disorder (15%). Twenty-three patients (12%) reported suicidal ideation at time of assessment.

**Table 3:** Prevalence of DSM-IV diagnoses and comorbidity and treatment engagement

Diagnoses (N = 195)	N (%)	
Any mental health diagnosis	162 (83)	
Mood disorders		
Any mood disorder	144 (74)	
Major depressive disorder	87 (45)	
Bipolar I and II disorders	30 (15)	
Dysthymic disorder	1 (< 1)	
Depressive disorder not otherwise specified	26 (13)	
Anxiety disorders		
Any anxiety disorder	67 (34)	
Posttraumatic stress disorder (PTSD)	29 (15)	
Generalized anxiety disorder	27 (14)	
Panic disorder	5 (3)	
Anxiety disorder not otherwise specified	7 (4)	
Psychotic disorders		
Any psychotic disorder	13 (7)	
Schizoaffective disorder	5 (3)	
Schizophrenia	3 (< 2)	
Delusional disorder	1 (< 1)	
Psychotic disorder not otherwise specified	4 (2)	
Adjustment disorder	1 (< 1)	
Suicidal ideation	23 (12)	
Personality disorder (any)	14 (7)	
Substance use disorders (N = 195)	Currently meet criteria	Ever met criteria
	N (%)	N (%)
Any substance use disorder	98 (50)	122 (63)
Alcohol	53 (27)	79 (41)
Amphetamine	1 (< 1)	1 (< 1)
Cannabis	39 (20)	48 (25)
Cocaine	46 (24)	62 (32)
Hallucinogen	1 (< 1)	3 (< 2)
Opioid	40 (21)	49 (25)
Sedative, Hypnotic, or Anxiolytic	7 (4)	7 (4)
Nicotine dependence	75 (38)	90 (46)
Mental Health/Substance use comorbidity (N = 195)	N (%)	
Mental Health only	78 (40)	
Active Substance Use Disorder only	14 (7)	
Mental Health and Active Substance Use Disorder	84 (43)	
No Mental Health or Active Substance Use Disorder	19 (10)	
Top 3 Mental Health comorbidities		
Major depressive disorder and generalized anxiety Major depressive disorder and PTSD Bipolar disorder and PTSD	19 (10) 12 (6) 11 (6)	
Engaged in treatment at time of assessment	No. (%)	
Mental health diagnosis (*N* = 162) and engaged in treatment	27 (17)	
Substance use diagnosis (*N* = 98) and engaged in treatment	10 (10)	

Note: Substance use disorders include both abuse and dependence. *Current criteria* includes only participants meeting criteria for abuse or dependence at time of assessment. *Ever met criteria* includes cases in remission. Percentages for Mental Health and Substance Use diagnoses do not add to 100 due to comorbidity

One-half (50%) of patients met criteria for a current substance use disorder. Including cases in remission, the most common substance was alcohol (41%), followed by cocaine (32%) and opioids (25%). However, only 10% of patients who met criteria for substance use disorder were engaged in treatment for their substance use disorder at the time of assessment.

Comorbidity among psychiatric disorders was observed in 41% of patients; major depressive disorder combined with generalized anxiety disorder was the most prevalent pairing. Thirty percent of patients had an active substance use disorder comorbidity; alcohol/marijuana and cocaine/opioids were the two most frequently occurring dyads. Four in ten (43%) patients had a psychiatric and substance use disorder comorbidity.

## Discussion

Several of our findings have implications for primary care delivery. We found that the vast majority of patients met criteria for a psychiatric and/or substance use disorder (90%), but few were engaged in treatment at the time of assessment; only 27 (17%) were engaged in mental health treatment, and of the 98 patients with active substance use disorder, 10 were engaged in substance use treatment. However, nearly all patients stated they had a PCP, most of whom indicated they had seen their provider within three months of their last hospital discharge.

These findings underscore the need for greater knowledge about the obstacles to providing integrated medical and behavioral health care in primary care settings, as well as the need for policies and incentives to better support primary care providers in their efforts to address the behavioral health needs of their patients. Obstacles to providing integrated care may include lack of coordination across medical and behavioral health care delivery, limited resources for behavioral health and substance use treatment, patient reluctance to discuss behavioral health issues with their primary care provider, financial and insurance coverage limitations, and challenges in navigating complex healthcare systems. The integration of primary care and behavioral health has garnered attention from researchers and practitioners in recent years, leading to the development of guides and recommendations on care integration [27–29]. Based on our findings, we recommend that primary care settings implement integrated care strategies, such as routinely assessing all patients for behavioral health needs (e.g. depression screening), identifying patients who would benefit from integrated care, embedding behavioral health providers within their primary care teams, and developing shared care plans and connections with other community agencies providing behavioral health and substance use treatment.

Our findings also provide support for implementing and improving targeted interventions for related disorders. We found that mood disorders are by far the most prevalent class of psychiatric disorders (74%), while alcohol, cocaine, and opioids are the three most common substances for substance use disorders. Similarly, we found a high degree of comorbidity among psychiatric and substance use disorders, highlighting the need for research into how psychiatric and substance use comorbidity affects patients’ ability to follow recommended medical treatment, which is a driver of avoidable hospital utilization [30]. Furthermore, given the mortality rates associated with psychiatric disorders, efforts are needed to quantify and address the role of specific psychiatric and substance use disorders in preventable mortality among patients with chronic medical conditions and with high hospital utilization. Given that patients with behavioral health conditions tend to die from their chronic medical conditions rather from their mental health condition(s), [9] future studies should also examine how specific chronic medical conditions are potentially relevant to understanding or managing patients’ psychiatric and/or substance use disorders.

Housing instability and a history of traumatic experiences were also prevalent among the patients in our study. Housing interventions that include supportive services have been effective in improving health outcomes [31, 32]. Primary care providers need the support of social service providers to refer patients to appropriate resources and address housing instability and other social determinants as factors affecting patients’ ability to participate fully in treatment.

Nearly two-thirds of patients in this study reported exposure to trauma during childhood, adulthood, or both. The high prevalence of trauma exposure among these patients highlight why it is imperative that health care providers understand the role that trauma plays in the lives of people they serve, and learn about strategies to promote patients’ comfort and engagement with the health care system. Exposure to trauma has been frequently linked with many medical and behavioral health conditions, including hypertension, [33] asthma, [34] depression, [35] and suicidality, [36] all of which were common characteristics in this patient population. The Substance Abuse and Mental Health Administration offers information about trauma-informed approaches and interventions [37] and trauma-informed primary care-specific initiatives have been developed, including *Trauma Informed Primary Care Initiative* [38] and *Advancing Trauma-Informed Care* [39]. However, to effectively implement these strategies, primary care offices need the support of policies and funding reforms that incentivize such approaches. While some state and federal legislation has been proposed or passed in recent years to encourage trauma-informed care, policymakers need to continue this trend by measuring the financial benefits of trauma-informed care and by implementing funding reforms that make implementation in health care settings financially viable [40].

### Limitations

Several factors limit the generalizability of our results. First, our findings may be most relevant to urban areas whose sociodemographic characteristics are most similar to Camden, New Jersey. Therefore, comparing our results to studies conducted in different locales must be done with caution. Second, patients enrolled in the Camden Coalition’s care management intervention experience patterns of high hospital use. The findings of our study may not be applicable to individuals experiencing high use of other health-related services. Third, while our data highlight important behavioral health characteristics within a population of patients with frequent hospital admissions, no conclusions can be drawn about an association between having a psychiatric and/or substance use disorder and frequent hospitalizations. A fourth limitation is that our results are based on behavioral health assessments of patients who were referred to a clinical psychologist by their care teams, as opposed to assessments for all patients enrolled in the Camden Coalition’s care management intervention. We do not know the extent to which our results represent the average individual enrolled in our intervention. Finally, because nearly all of these patients were covered under Medicaid and/or Medicare insurance, our findings cannot be compared to patients with other types of health insurance coverage or no coverage at all.

## Conclusions

Our findings may be most relevant for providers and researchers whose work addressees the complex health and social needs of patient or client populations and the behavioral correlates of health and disease. Specifically for primary care providers, this study highlights the: (1) importance of identifying, treating, and referring patients to proper treatment to address patients’ psychiatric and substance use disorders; (2) need for targeted primary care efforts specifically for mood disorders and alcohol, cocaine, and opioid related disorders; (3) need to identify and address non-medical barriers to treatment such as housing instability; and (4) necessity of trauma-informed integrated physical and behavioral health care.

## Abbreviations

Camden Coalition: The Camden Coalition of Healthcare Providers
DSM-IV: Diagnostic and Statistical Manual of Mental Disorders
PCP: primary care provider
